# Metastatic Acetabular Fracture: A Rare Disease Presentation of Recurrent Head and Neck Paraganglioma

**DOI:** 10.7759/cureus.7596

**Published:** 2020-04-09

**Authors:** Tye Patchana, Juston Fan, Max Jiganti, Ronaldo D Gnass

**Affiliations:** 1 Neurosurgery, Riverside University Health System Medical Center, Moreno Valley, USA; 2 Orthopaedic Surgery, Riverside University Health System Medical Center, Moreno Valley, USA; 3 Orthopaedic Surgery, Children's Hospital of Orange County, Orange County, USA; 4 Orthopaedic Surgery, Burrell College of Osteopathic Medicine, Las Cruces, USA; 5 Pathology, Riverside University Health System Medical Center, Moreno Valley, USA

**Keywords:** paraganglioma, carotid body tumor

## Abstract

We present a case of a rare metastatic bone lesion of the acetabulum, associated with a pathologic fracture, found to be metastasis from a malignant carotid body paraganglioma upon histological analysis. We present a report of the patient’s clinical course following the identification of metastatic disease to the right acetabulum, as well as a review of paragangliomas and their propensity for metastasis.

## Introduction

Paragangliomas are rare tumors of neuroendocrine cells from autonomic paraganglia outside the adrenal glands. A distinction is made from intra-adrenal endocrine tumors, defined as pheochromocytomas, that are distinguished by adrenergic phenotypes, lower rates of malignancy, and a stronger association with hereditary phenotypes [1]. Paraganglioma tumors can be seen in the sympathetic chain ganglia associated with catecholamine secretion, but can also be found in the suboccipital and neck region in parasympathetic ganglia, which are often asymptomatic [2]. The high vascularity of these tumors cause them to have a predilection for the carotid body, and the histological analysis often reveals excess catecholamine metabolites, primarily norepinephrine [3-5]. Excess catecholamine secretion is also seen in pheochromocytoma, an intra-adrenal neoplasm that mirrors symptoms of paragangliomas and can be difficult to distinguish [6]. The classic presentation typically occurs as a painless neck mass. Cranial nerve deficits may also be present.

## Case presentation

A 28-year-old male presented to the emergency department (ED) with a chief complaint of right hip pain following a restrained motor vehicle accident (MVA). The patient had a past medical history significant for resection of right carotid body paraganglioma approximately ten years prior. At that time, the patient presented to the primary care clinic with complaints of severe generalized headaches, syncopal episodes, and associated blurry vision. Initial complaints of non-tender, right-sided neck mass resulted in a biopsy, at that time diagnosed as a benign lymph node. Upon continued symptoms, he was eventually referred to the oncology clinic for follow up for his neck mass. After a subsequent biopsy of the lesion, a carotid body paraganglioma was diagnosed and excised, and he was treated with two months of radiation without chemotherapy.

**Figure 1:**
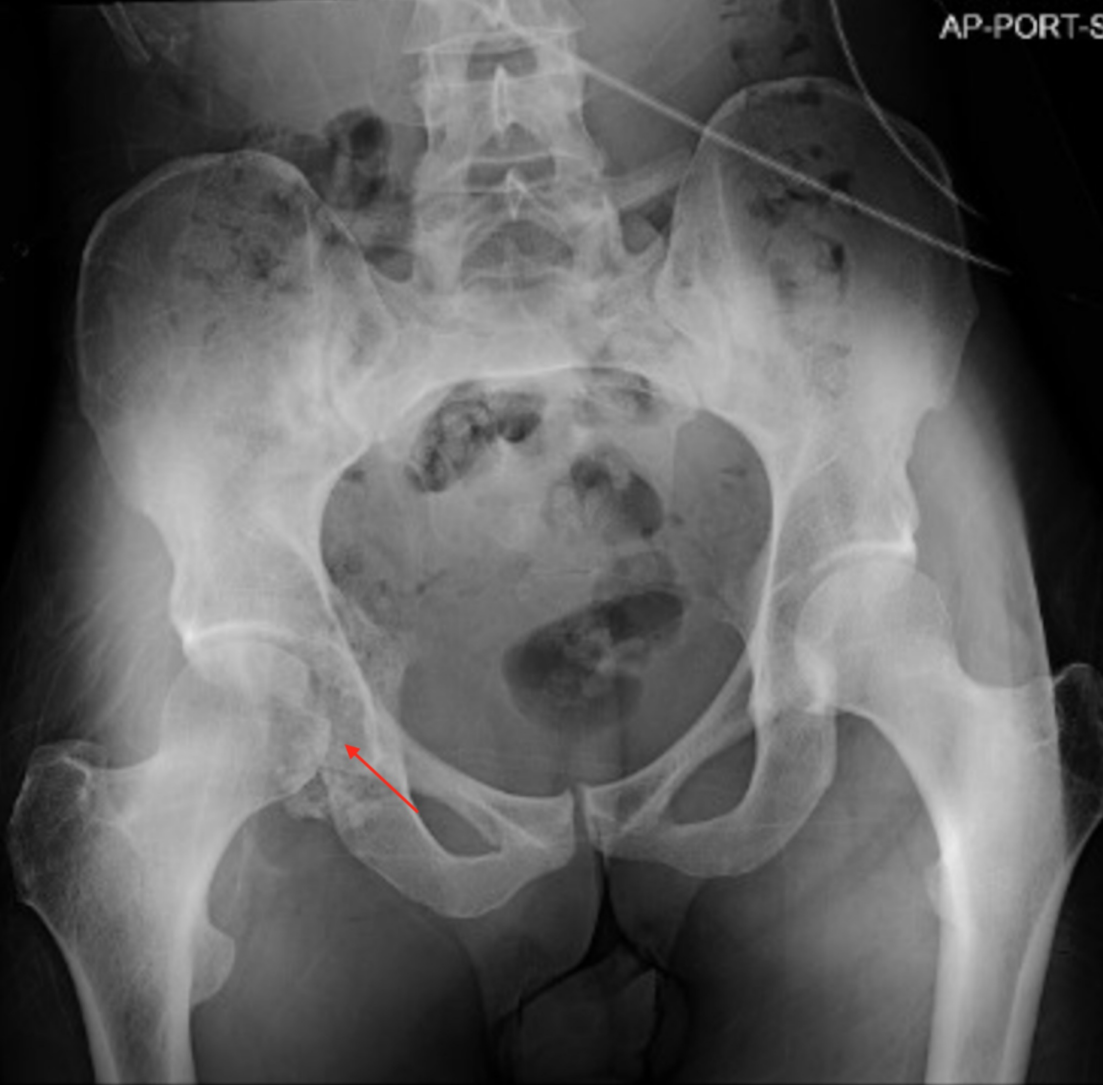
Anterior-posterior X-ray of the pelvis demonstrating lucency and bone destruction associated with the right acetabulum, suspicious for metastatic disease

**Figure 2:**
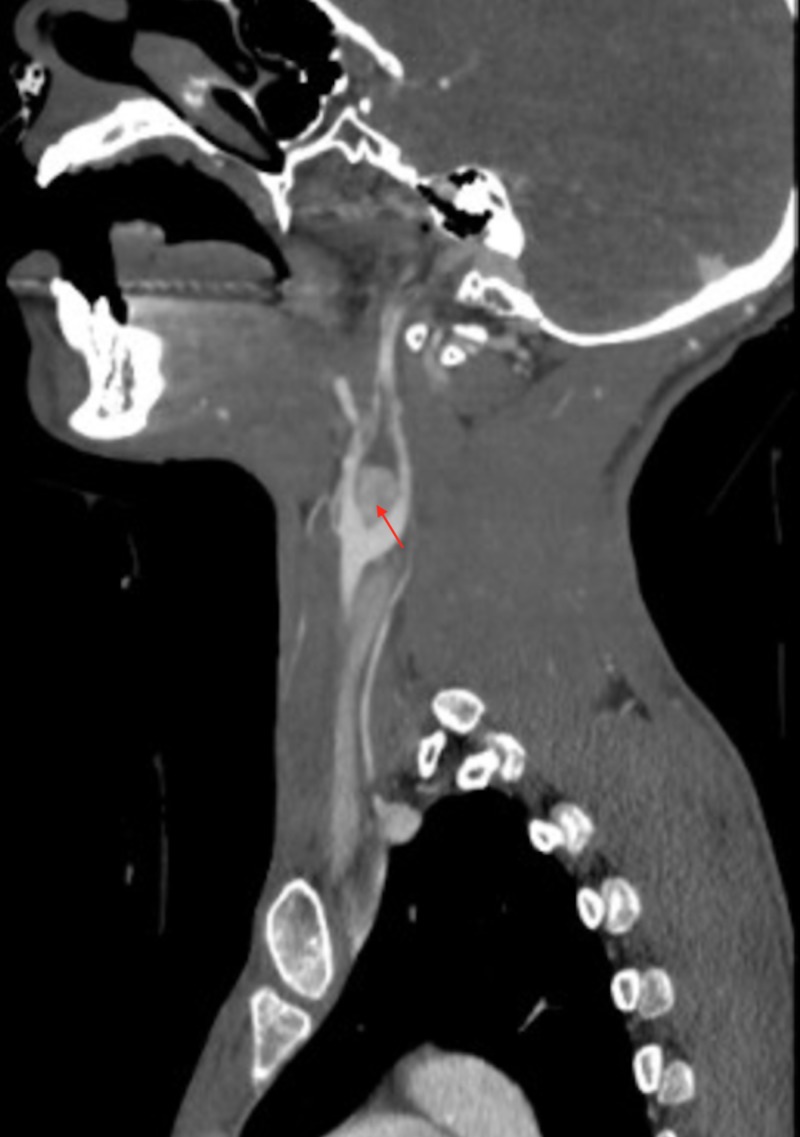
Sagittal view of computed tomography angiogram of the neck demonstrating an enhancing mass, approximately 13 mm in diameter, splaying the left internal and external carotid artery, consistent with paraganglioma

**Figure 3:**
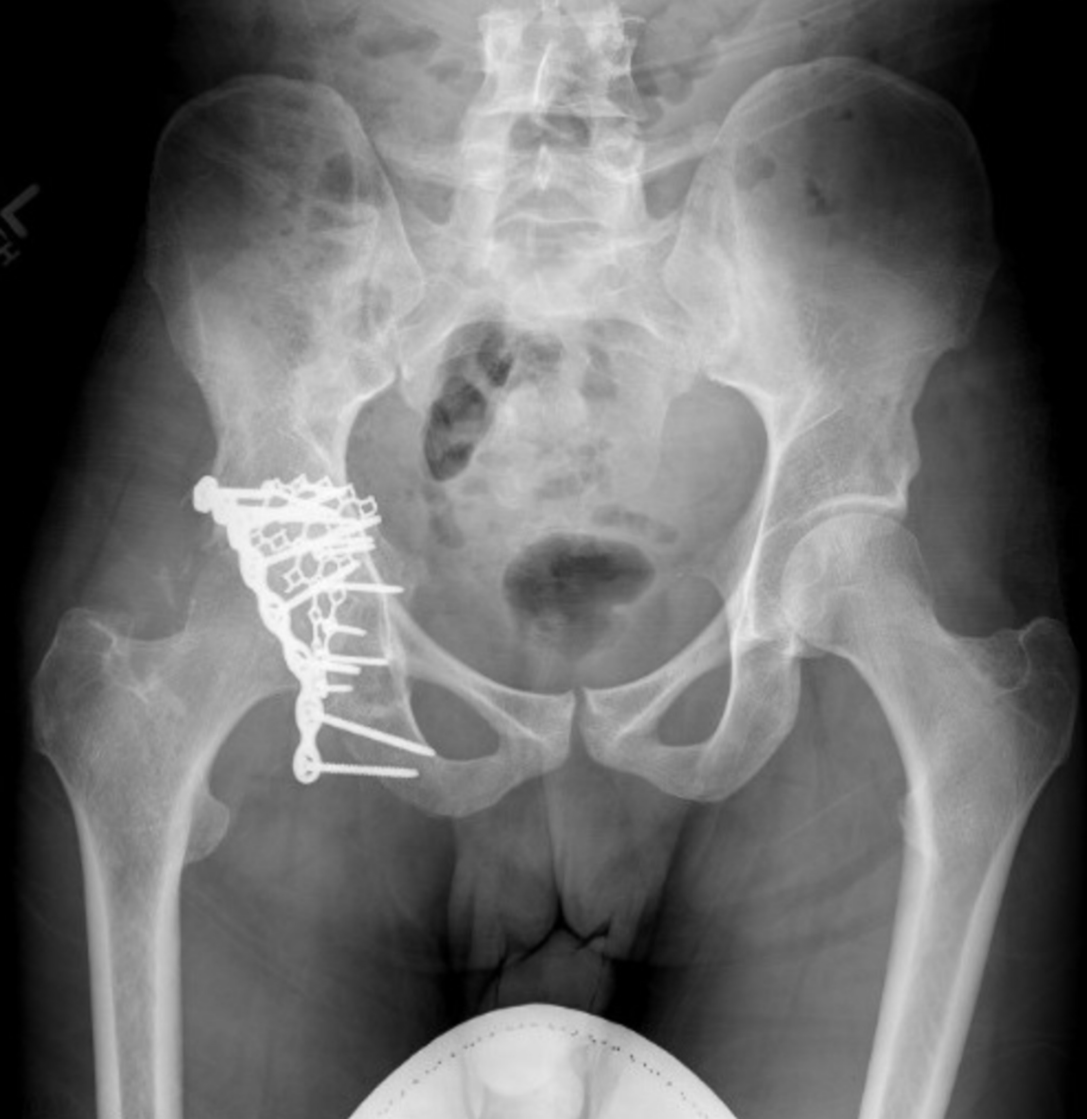
Anterior posterior X-ray of the pelvis demonstrating fixation of the right acetabulum following open reduction internal fixation and bone biopsy

**Figure 4:**
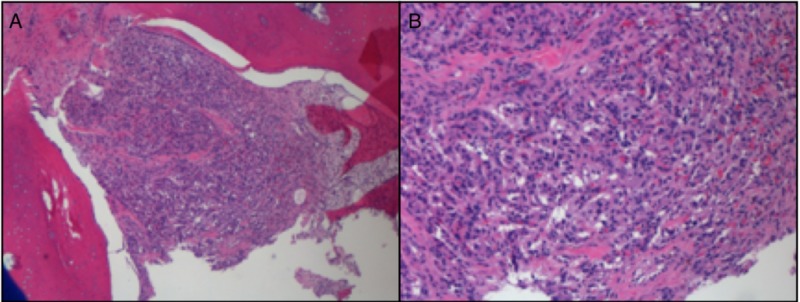
A) Hematoxylin and eosin (H&E) stain demonstrating bone infiltrated by nests of round to oval epithelioid cells, surrounded by vascular septae at 40x magnification; B) H&E stain demonstrating the tumor cells show abundant eosinophilic to clear cytoplasm and uniform nuclei at 100x magnification

**Figure 5:**
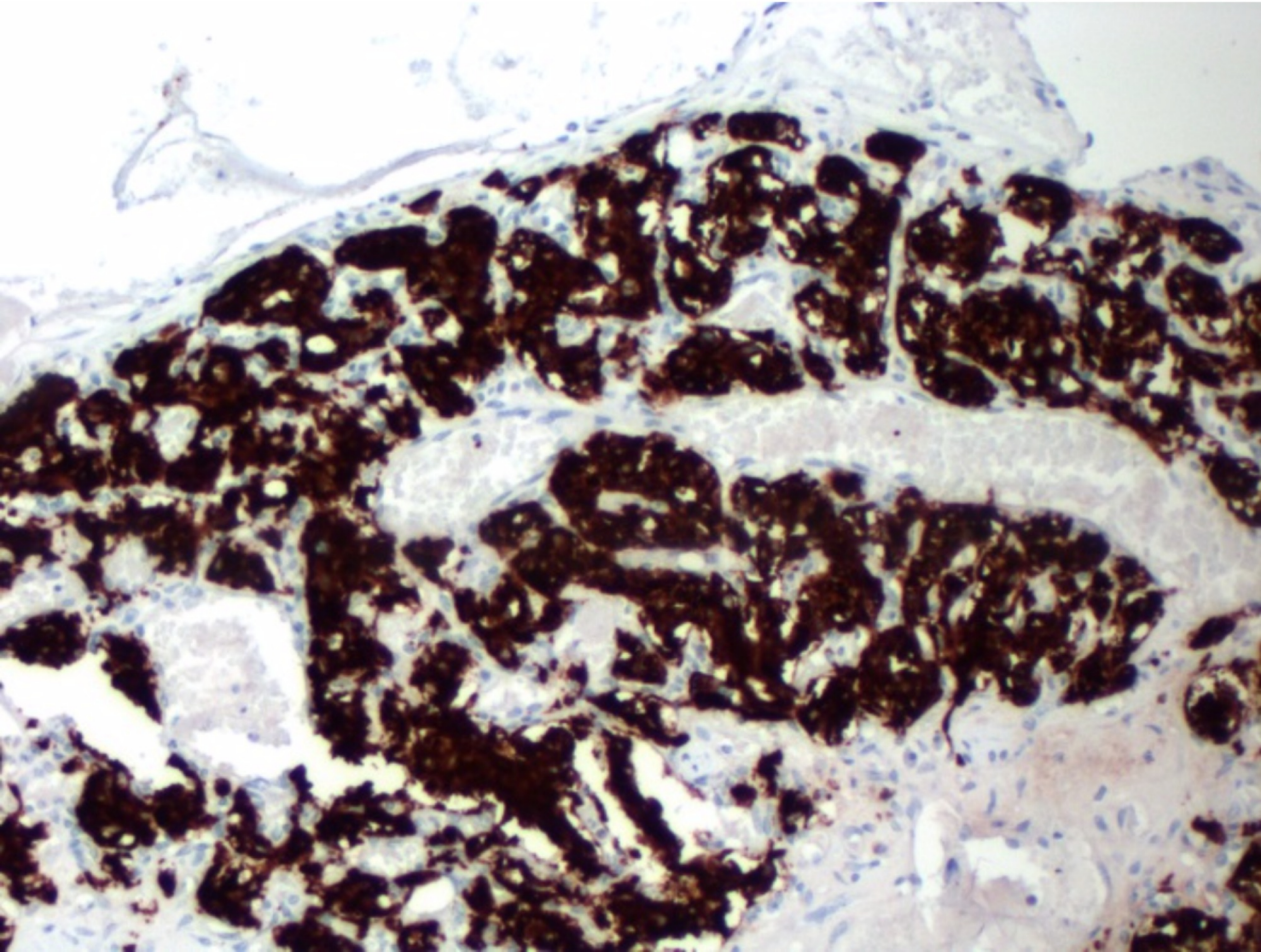
Tumor cells demonstrating intense positivity with synaptophysin at 100x magnification

**Figure 6:**
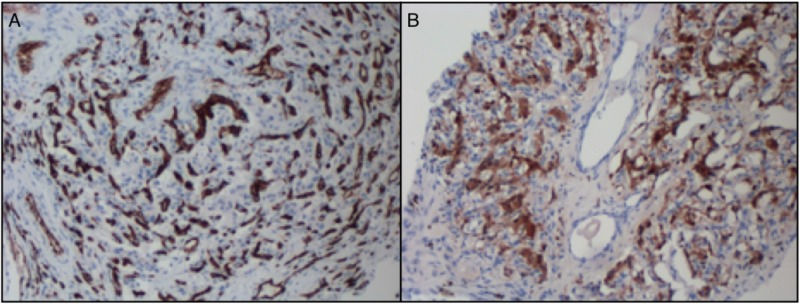
A) S100 immunostain highlights sustentacular cells at 100x magnification; B) CD34 immunostain highlights vascular channels at 100x magnification CD34: cluster of differentiation 34.

**Figure 7:**
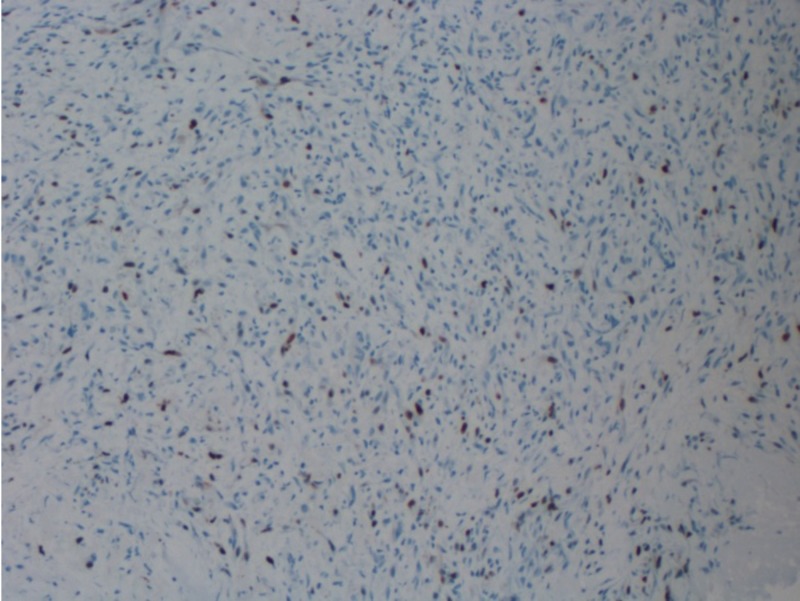
Ki-67 immunostain (100x); proliferation index is 1%-4% A proliferative labeling index of Ki-67 >3% is considered a useful parameter for predicting malignant potential.

Upon presentation to the ED, a pathologic acetabular fracture was discovered. On physical exam, the patient demonstrated tenderness to palpation of the right hip. He noted recent night sweats, as well as weight loss. Anteroposterior (AP) X-rays of the pelvis demonstrated a right acetabular lytic lesion (Figure 1). Additionally, computed tomography angiogram (CTA) of the neck demonstrated a left carotid body lesion, approximately 13 mm in diameter splaying the left internal and external carotid artery, consistent with paraganglioma (Figure 2). The patient underwent open reduction internal fixation (ORIF) to the right pathological posterior wall acetabulum fracture and biopsy of bone marrow. The bone marrow biopsy demonstrated the presence of infiltration from a metastatic malignant paraganglioma via histopathological analysis (Figures 3-7).

## Discussion

Accounting for approximately 0.3% of all neoplasms, paragangliomas are rare tumors arising from the extra-adrenal paraganglia [7]. The rarity of metastatic paragangliomas was exhibited by a National Cancer Database study identifying only 10 cases over a 10-year period (1985-1996) [8]. We present a case of a 28-year-old male with a history of carotid body paraganglioma presenting with recurrence and metastasis to the femur. CTA of the neck, as seen in Figure 2, demonstrates a classical sign, Lyre sign, associated with carotid body tumors, typified by splaying of the internal and external carotid arteries [9].

Pheochromocytomas and extra-adrenal paragangliomas share identical histological morphology. However, a distinction between the two is important as the latter have a greater propensity for malignancy, less adrenergic symptoms, and is not as strongly associated with hereditary syndromes [1]. Hematoxylin and eosin (H&E) staining demonstrated a classical pattern associated with paraganglioma, as seen in Figure 3. Previous studies have demonstrated a Ki index > 3% to be indicative of metastatic potential [10,11]. Staining for Ki-67 from this patient, upon pathological examination of bone biopsy, demonstrated a Ki-67 index between 1%-4%.

Metastasis of paragangliomas is a proportionally rare event, with only 10-30% of cases advancing to metastatic disease, depending on the study parameters [12,13]. Accordingly, attempts have been made to delineate the histopathological features predictive of metastasis. Though the majority of cases are surgically resectable, those that advance to a malignant state have an intractable clinical course. Secondary to long latency periods between the discovery of primary tumor and metastasis, patients suspected of having metastatic disease require lifelong surveillance [14]. Previous studies have demonstrated the mean time to recurrence after initial resection to be approximately 3-6 years [10,15]. Our patient underwent an uncomplicated clinical course following ORIF of the right acetabulum, with discharge instructions for follow up with the orthopedic clinic, otolaryngology clinic, and hematology/oncology for future imaging surveillance.

## Conclusions

We present a rare case of a 28-year-old male with a history of carotid body paraganglioma resection, who presented to the ED with a chief complaint of right hip pain, following a restrained MVA. Subsequent imaging demonstrated a pathological fracture of the acetabulum with imaging suspicious for metastatic disease to the bone. The patient subsequently underwent ORIF to the right pathological posterior wall acetabulum fracture and biopsy of bone marrow, demonstrating the presence of infiltration from a metastatic malignant paraganglioma. This case highlights some of the features of metastatic paragangliomas with a discussion of the propensity for metastasis, as well as the histopathological analysis. Patients found to have metastatic paragangliomas will require life-long surveillance.
